# Suicidal ideation and suicide attempt following ketamine prescription in patients with treatment-resistant depression: a nation-wide cohort study

**DOI:** 10.21203/rs.3.rs-3207199/v1

**Published:** 2023-08-07

**Authors:** Yiheng Pan, Maria P. Gorenflo, Pamela B. Davis, David C. Kaelber, Susan De Luca, Rong Xu

**Affiliations:** 1 Center for Artificial Intelligence in Drug Discovery, Case Western Reserve University School of Medicine, Cleveland, OH, USA; 2 Department of Computer and Data Science, Case Western Reserve University, Cleveland, OH, USA; 3 Center for Community Health Integration, Case Western Reserve University School of Medicine, Cleveland, OH, USA; 4 The Center for Clinical Informatics Research and Education, The MetroHealth System, Cleveland, OH, USA; 5 Population Health Research Institute, The MetroHealth System, Cleveland, OH, USA

## Abstract

Ketamine, including esketamine, is an effective treatment for patients with treatment-resistant depression (TRD); however, its long-term efficacy in real-world populations remains poorly characterized. This is a retrospective cohort study using TriNetX US Collaborative Network, a platform aggregating electronic health records (EHRs) data from 93 million patients from 56 health care organizations in the US, and the study population includes 321,367 patients with a diagnosis of TRD who were prescribed relevant treatment in their EHRs. The prescription of ketamine (including esketamine) was associated with significant decreased risk of suicidal ideation compared to prescription of other common antidepressants: HR = 0.65 (95% CI: 0.53 – 0.81) at 1 day – 7 days, 0.78 (95% CI: 0.66 – 0.92) at 1 day – 30 days, 0.81 (95% CI: 0.70 – 0.92) at 1 day – 90 days, 0.82 (95% CI: 0.72 – 0.92) at 1 day – 180 days, and 0.83 (95% CI: 0.74 – 0.93) at 1 day – 270 days. This trend was especially robust among adults over 24 years of age, males, and White patients with TRD. No significant difference was observed for suicide attempts, except significantly increased risk for adolescents (aged 10-24) at 1 day – 30 days with HR = 2.22 (95% CI: 1.01-4.87). This study provides real-world evidence that ketamine has long-term benefits in mitigating suicidal ideation in patients with treatment-resistant depression. Future work should focus on optimizing dosage regimens for ketamine, understanding the mechanism, and the difference in various demographic subpopulations.

## Introduction

Ketamine, a Food and Drug Administration (FDA)-approved anesthetic, and its S enantiomer S-ketamine (esketamine), provide well-characterized antidepressant benefits, rapidly and transiently alleviating manifestations of treatment-resistant depression (TRD), including suicidal ideation^1-7^. Although ketamine and esketamine are not identical, they share a mechanism of action in that both antagonize N-methyl D-aspartate receptors^4,8^. The benefits of both drugs for TRD and suicidal ideation have been demonstrated in clinical trials for up to one month after drug administration, but evidence of longer-term benefits remains limited^9-16^. Additionally, there is evidence that both drugs are associated with reduction of suicidal ideation, but the risk of actual suicidal behavior remains unknown^6^.

Suicide is a leading cause of death in the United States (and is the second leading cause of death among 10-34 year-olds and fifth leading cause of death among 35-54 year-olds)^17^, and was associated with over 45,000 deaths in 2021 alone^18^. In the same year, an estimated 12.3 million American adults had suicidal thoughts, and 1.7 million attempted suicide^18^. Thirty percent of patients with TRD attempt suicide at least once during their lifetime^19-21^. In 2019, the FDA approved esketamine nasal spray for the treatment of TRD^24^; however, ketamine is not FDA-approved for the treatment of any psychiatric disorder^8^.

In this large-scale retrospective cohort study, we assessed the risk of suicidal ideation and attempted suicide among TRD patients prescribed ketamine (including esketamine) compared to those prescribed other antidepressants and how the risk evolves over time from 7 days to 270 days. Since risk of suicide ideations and behaviors varies by age, gender and race^22,23^, we also conducted stratified analyses to examine these effects in different demographic subgroups.

## Methods

### (1) Database Description

The TriNetX Analytics Network platform is a large-scale, deidentified database. Data were obtained from the US Collaborative Network, which consists of 93 million unique patients from 56 healthcare organizations, covering diverse geographic locations, age groups, race and ethnic groups, income levels, and insurance types^25^. Built-in statistical functions within the TriNetX Analytics Platform perform statistical analyses on patient-level data and report population-level results without including protected health information. The Institutional Review Board of the MetroHealth System, Cleveland, Ohio has determined that any research using TriNetX in this manner is not Human Subject Research and is therefore exempt from IRB review. We have previously used TriNetX to conduct retrospective cohort studies^26-39^. This study follows the STROBE (Strengthening the Reporting of Observational Studies in Epidemiology) reporting guidelines^40^.

### (2) Study population

**Figure 1.**
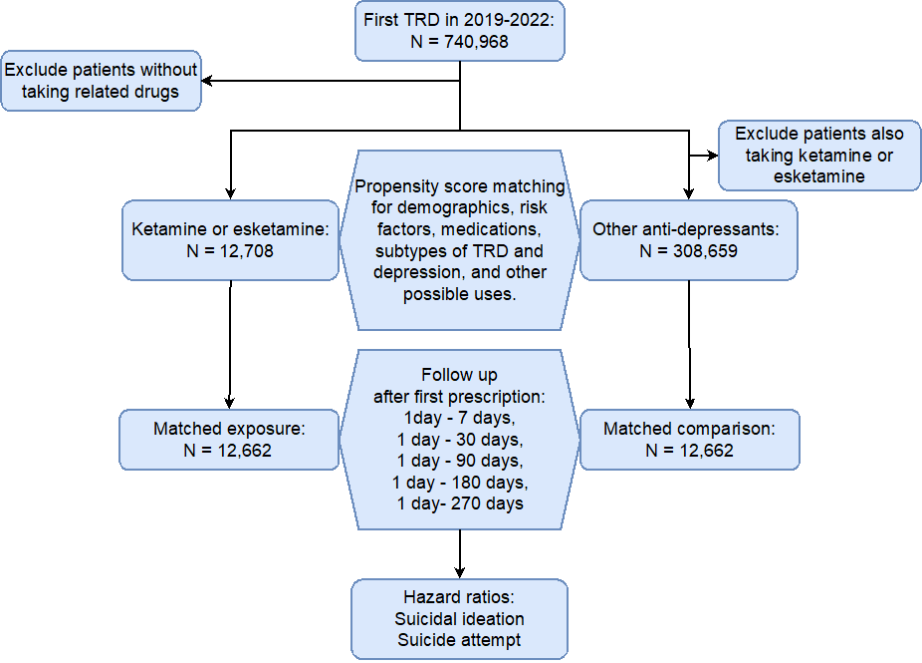
Flow diagram of patient selection and analysis in TriNetX. (TRD: treatment resistant depression)

The study population consisted of patients who had their first encounter diagnosis of TRD and were followed by the prescription of related treatments between January 2019 and January 2022. To compare the prescription of ketamine (including esketamine) with that of other common antidepressants, the study population was divided into two cohorts: (1) an exposure cohort consisting of TRD patients who were prescribed ketamine (or esketamine), we will use “ketamine” through this paper to refer ketamine or esketamine, and (2) a comparison cohort consisting of TRD patients who were not prescribed ketamine but rather at least one of the following antidepressants: fluoxetine, paroxetine, sertraline, citalopram, escitalopram, vortioxetine, venlafaxine, duloxetine, doxepin, amitriptyline, trazodone, mirtazapine, or bupropion. The study population excluded patients not taking antidepressants as they may display relatively mild symptoms of depression or be in remission from TRD (Figure 1). Importantly, patients in the exposure cohort could be prescribed other antidepressants in addition to ketamine, as they are often prescribed in conjunction with other treatments^41^. Prescription of the non-ketamine antidepressants listed above was therefore balanced between the two cohorts using propensity-score matching.

### (3) Statistical analysis

The outcomes of interest were one or more encounter diagnoses of suicidal ideation (ICD-10: R45.851 Suicidal ideation) and suicide attempt (ICD-10: T14.91 Suicide attempt).

Covariates that were matched between the exposure and comparison cohorts include demographics (age, gender, race, and ethnicity), and potential confounders^5,42^ including pre-existing medical conditions, medications, procedures, family history, and socioeconomical factors (Table 1). The full list of outcomes and covariates, as well as their standardized names, data types, and corresponding codes, are included in **eTables 1** and **2**.

Statistical analyses were conducted in the TriNetX Advanced Analytics platform. Cohorts were propensity-score matched (1:1 matching using the nearest neighbor greedy matching algorithm) for the above covariates. The index event for the exposure and comparison cohorts was the first prescription of either ketamine or other antidepressant after TRD diagnosis. Hazard ratios of the outcomes of interest at 1 day – 7 days, 1 day −30 days, 1 day – 90 days, 1 day – 180 days, and 1 day – 270 days after drug prescription were compared between matched cohorts using hazard ratios (HRs) and 95% confidence intervals (CIs), and p-values were calculated at a significance of P < 0.05 (2-sided t-test)^43^ (Figure 1).

As suicidal thoughts and behaviors vary by age, gender, and race^18^, stratified analyses were performed in subgroups distinguished by age (adolescents [10-24 years old]^44,45^, adults [>=24 years old])^46^, gender (female, male)^22^ and race (White, Black)^47^. Due to the relatively small sample size, stratified analyses were not performed for other races. Figure 1 presents the flow diagram of patient selection and analysis in TriNetX.

## Results

### (1) Patient characteristics

Table 1 and **eTable 3** present the characteristics of the patients in the exposure cohort (those prescribed ketamine) and the comparison cohort (those prescribed other antidepressants) before and after propensity-score matching. Before matching, the exposure cohort was older (average 49.4 vs. 43.2 years) and had significantly higher prevalence of comorbidities and adverse socioeconomic determinants of health. The two cohorts were balanced after propensity-score matching, yielding 12,662 patients each in the exposure and comparison cohorts (Table 1).

### (2) Ketamine prescription is associated with decreased suicidal ideation compared to other antidepressant prescription.

**Figure 2.**
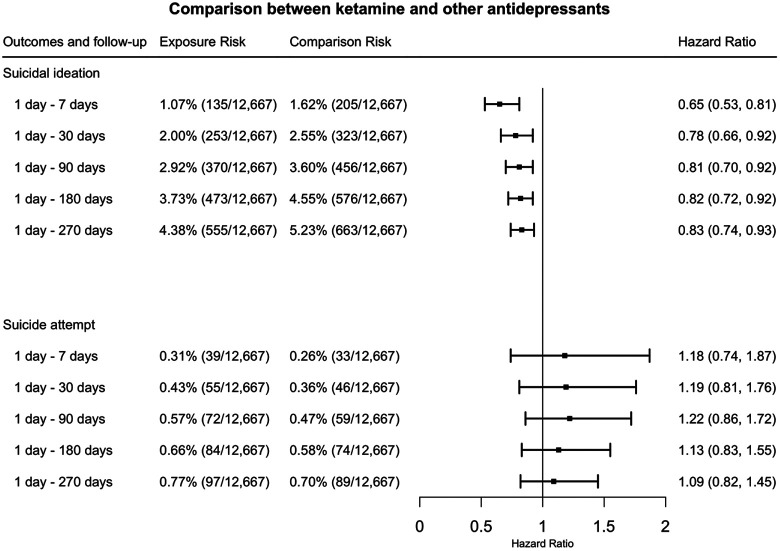
Comparison of hazard of suicidal ideation and suicide attempt among patients with TRD between propensity-score matched ketamine cohort and the comparison cohort (other anti-depressants) at 1day – 7 days, 1 day −30 days, 1 day – 90 days, 1 day – 180 days, 1 day – 270 days after initial prescription. (TRD: treatment resistant depression)

As shown in Figure 2, the prescription of ketamine was associated with significant decrease in suicidal ideation: HR = 0.65 (95% CI: 0.53 – 0.81) at 1 day – 7 days, 0.78 (95% CI: 0.66 – 0.92) at 1 day – 30 days, 0.81 (95% CI: 0.70 – 0.92) at 1 day – 90 days, 0.82 (95% CI: 0.72 – 0.92) at 1 day – 180 days, 0.83 (95% CI: 0.74 – 0.93) at 1 day – 270 days. No significant difference was observed for suicide attempt.

### (3) The association between ketamine prescription and suicidal ideation or suicide attempt varies by age, gender, and race.

**Figure 3.**
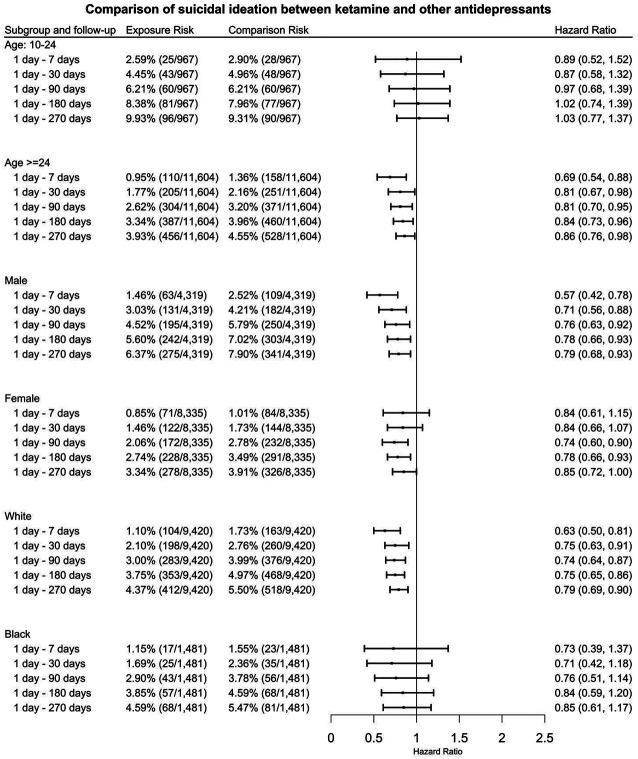
Comparison of hazard of suicidal ideation among patients with TRD (matched ketamine cohort vs. other anti-depressants cohort) stratified by age, gender, race groups at 1day – 7 days, 1 day −30 days, 1 day – 90 days, 1 day – 180 days, 1 day – 270 days after initial prescription. (TRD: treatment resistant depression)

In patients ages 24 and older, ketamine prescription is associated with significant decrease in suicidal ideation compared to prescription of other antidepressants at 1 day – 7 days, 1 day −30 days, 1 day – 90 days, 1 day – 180 days, 1 day – 270 days after initial prescription: HR = 0.69 (95% CI: 0.54 – 0.88), 0.81 (95% CI: 0.67 – 0.98), 0.81 (95% CI: 0.70 – 0.95), 0.84 (95% CI: 0.73 - 0.96), 0.86 (95% CI: 0.76-0.98) (Figure 3). In male patients, ketamine prescription is associated with significant decrease in suicidal ideation compared to prescription of other antidepressants at 1 day – 7 days, 1 day −30 days, 1 day – 90 days, 1 day – 180 days, 1 day – 270 days after initial prescription: HR = 0.57 (95% CI: 0.42 – 0.78), 0.71 (95% CI: 0.56 – 0.88), 0.76 (95% CI: 0.63 – 0.92), 0.78 (95% CI: 0.66 - 0.93), 0.79 (95% CI: 0.68-0.93) (Figure 3). In White patients, the prescription of ketamine is associated with significant decrease in suicidal ideation compared to prescription of other common antidepressants at 1 day – 7 days, 1 day −30 days, 1 day – 90 days, 1 day – 180 days, 1 day – 270 days after initial prescription: HR = 0.63 (95% CI: 0.50 – 0.81), 0.75 (95% CI: 0.63 – 0.91), 0.74 (95% CI: 0.64 – 0.87), 0.75 (95% CI: 0.65 - 0.86), 0.79 (95% CI: 0.69-0.90) (Figure 3). In female patients, the prescription of ketamine is associated with significant decrease in suicidal ideation compared to prescription of other common antidepressants at 1 day – 90 days, 1 day – 180 days, 1 day – 270 days after initial prescription: HR = 0.74 (95% CI: 0.60 – 0.90), 0.78 (95% CI: 0.66 – 0.93), 0.85 (95% CI: 0.72 – 1.00). However, no significant differences were observed at 1 day – 7 days or 1 day – 30 days (Figure 3). No significant differences were observed for risk of suicidal ideation between the exposure and comparison cohorts in adolescents (aged 10-24 years) or Black patients.

**Figure 4.**
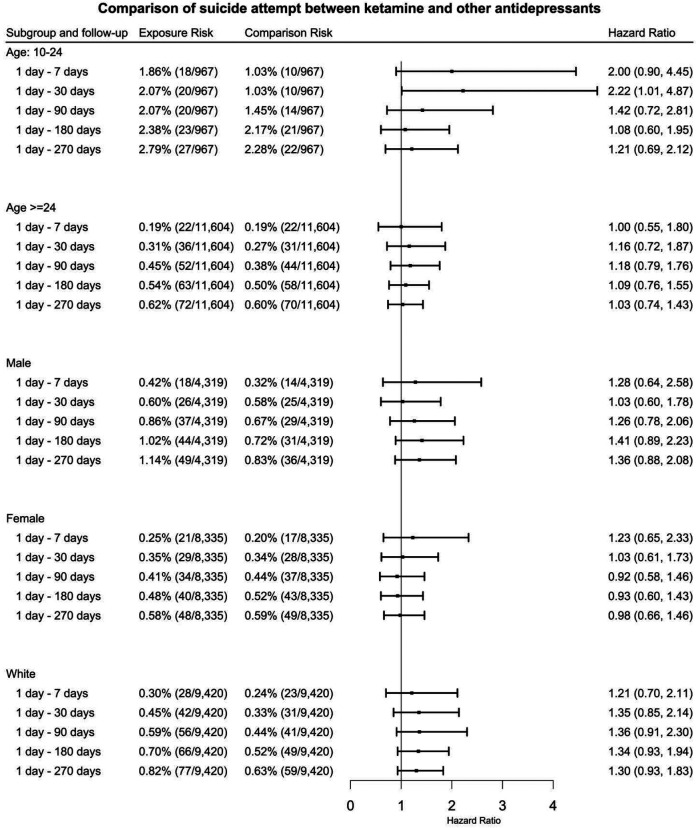
Comparison of hazard of suicide attempt among patients with TRD (matched ketamine cohort vs. other anti-depressants cohort) stratified by age, gender, race groups at 1day – 7 days, 1 day −30 days, 1 day – 90 days, 1 day – 180 days, 1 day – 270 days after initial prescription. (TRD: treatment resistant depression)

In patients aged 10-24, ketamine prescription is associated with increased risk of suicide attempt at 1 day – 30 days: HR = 2.22 (95% CI: 1.01-4.87). No other significant differences were observed for suicide attempt among the demographic-stratified subgroups (Figure 4), and the results for Black patients are not presented due to insufficient sample size.

## Discussion

Using a large-scale platform of aggregated patient electronic health record data, our study reveals that prescription of ketamine is associated with significant decrease in suicidal ideation in both the short- (1-30 days) and long-term (1-270 days) compared to other common antidepressants, and this trend was especially robust in those who were >=24 years of age, male, and White; however, no difference was observed in suicide attempt at any time point, except increased risk for adolescents (aged 10-24) at 1 day – 30 days. Randomized controlled trials have demonstrated that ketamine and esketamine can mitigate suicidal ideation in the short-term (one week, one month)^2,3,48-50^, consistent with our findings. This study also provides evidence of a long-term association between ketamine prescription and decrease in suicidal ideation in patients with TRD. This is also the first observational study to our knowledge that examines the risk over time of attempted suicide in patients with TRD who were prescribed ketamine versus other antidepressants.

In our results, decreased suicidal ideation does not translate into decreased suicide attempts. It has been recognized that suicidal ideation and suicide attempt have different mechanisms according to the ideation-to-action framework^51,52^, and there is no linear relationship between them^53^. Findings in stratified analyses suggest the decreased risk of suicidal ideation observed in those prescribed ketamine is especially strong among White, men, and over 24 years of age with TRD, which is possibly contributed by factors such as hormonal differences^54^, brain development^44^ and placebo effect^55^.

This study has several limitations. First, the results only represent individuals who had medical encounters with health care systems that provide data to TriNetX. Therefore, their generalizability to other populations needs to be further tested. Second, TriNetX only identifies the event of drug prescription, not the length of prescription or adherence to medication regimens. Since ketamine is potentially neurotoxic^56^, particularly with longer-term administration, clinicians typically prescribe ketamine for only a short period of time, after which patients are transitioned to maintenance treatment with antidepressants and/or psychotherapy^57^. Though our retrospective cohort study could not incorporate the duration of drug use or the specific antidepressants the exposure cohort switched to after the initial therapy of ketamine, the reduced effect of suicidal ideation persisted at 270 days post-prescription, indicating potential long-term benefits after pharmacotherapy transition. Third, we balanced the exposure and comparison groups using extensive propensity-score matching for demographics, risk factors, complications, and other treatments; however, there could be unmeasured confounders that have skewed the results. Fourth, due to the nature of retrospective cohort studies, our results cannot establish causality between ketamine prescription and reduction in suicidal ideation and cannot be used to impute the mechanism. Fifth, we utilized diagnosis of suicide attempt as a proxy for suicidal behavior, and TriNetX only includes the information that is entered during patients’ encounters with health organizations. Consequently, other suicidal behaviors that occurred outside of patient medical encounters may not be captured, resulting in missing outcome data and potential bias. Sixth, sufficient dosage data are not available on TriNetX, so it could not be determined if the relationship between ketamine prescription and suicidal ideation is dose dependent.

Future work should focus on optimizing dosage regimens for ketamine when it is used to treat TRD. The benefit and risk profile for esketamine has been established, as described in FDA-approved labelling^58^ and the approved Risk Evaluation and Mitigation Strategy program^24^. However, there is no FDA-approved dosing regimen for ketamine. Among the 740 968 patients from TriNetX platform who had first diagnosis of TRD during 2019-2022, there were 12,404 patients taking ketamine and 728 patients taking esketamine after their diagnosis. Considering the different dosing regimens, increasing popularity of ketamine for TRD, and potential adverse events related to ketamine prescription^59^, it is crucial to determine safe and effective dosing for ketamine when indicated for TRD^8^. In this study, we combined ketamine and esketamine together because of their similar mechanism of action and insufficient sample size in esketamine. A head-to-head comparison between ketamine and esketamine is necessary to examine the difference of these two drugs in the future. It is also important to further understand the underlying mechanism of ketamine effect among patients with TRD. One possible explanation is that ketamine can cause dissociative effects linked to an antidepressant response^60^. However, a recent clinical trial indicated that administration of ketamine under general anesthesia is no better than placebo at alleviating depression in the short term, and had similar effect to the previous ketamine trials in awake patients, suggesting that the drug may work through a patient’s interactions with medical professionals and a belief in improvement, rather than the biochemical effect of ketamine per se^61^. Further work is also needed to understand why the association between ketamine prescription with suicidal ideation and suicide attempt varies between different demographic subpopulations. It is worrisome that ketamine prescription did not mitigate suicidal ideation in adolescents and even was associated with increased trend of suicide attempt, which suggests the cautious prescription in this subpopulation. Future work is necessary to be conducted on larger sample size of adolescents.

## Conclusion

Our study provides real-world evidence that patients with TRD who were prescribed ketamine experienced significant long-term decrease in suicidal ideation compared with patients who were prescribed other antidepressants, within 270 days following the prescription. Findings from this study provide data to balance the benefits of ketamine with its reported adverse effects, such as dissociation, psychosis, hypertension, tachycardia, tolerance, and addiction^56,59,62^. Future work should focus on longer follow-up time, optimized dosage regimens for ketamine, its mechanism of action with respect to TRD and suicidal ideation, and disparities in efficacy between various demographic subgroups.

## Figures and Tables

**Table 1.** The characteristics of patients prescribed ketamine (“Ketamine cohort”) and other antidepressants (“Comparison cohort”) before and after propensity-score matching (SMD: standardized mean differences, *SMD greater than 0.1, a threshold being recommended for declaring imbalance.)

Characteristics	Before matching			After matching		
	Cohort, No. (%)			Cohort, No. (%)		
	Ketamine cohort	Comparison cohort	SMD	Ketamine cohort	Comparison cohort	SMD
Cohort size	12,708	308,659		12,662	12,662	
Age at Index	49.4 ± 16.7	43.2 ± 20.1	0.33*	49.4 ± 16.7	50.2 ± 19.1	0.03
Female	65.8	69.0	0.07	65.8	65.8	<.001
Race and ethnicity						
White	74.5	72.8	0.04	74.4	74.4	<.001
Unknown Race	12.2	13.7	0.05	12.2	12.5	0.01
Black or African American	11.8	11.2	0.02	11.8	11.4	0.01
Hispanic or Latino	6.2	7.5	0.05	6.2	6.1	0.01
Asian	0.8	1.6	0.08	0.8	0.8	0.01
Risk factors and complications (based on encounter diagnosis International Classification of Diseases (ICD) codes)						
Potential health hazards related to family and personal history	85.6	57.3	0.66*	85.5	86.7	0.03
Anxiety disorder, unspecified	59.9	44.9	0.31*	59.8	60.7	0.02
Hypertensive diseases	56.7	33.3	0.48*	56.6	57.1	0.01
Sleep disorders	49.9	28.3	0.45*	49.8	50.1	0.01
Chronic pain, not elsewhere classified	47.3	19.9	0.61*	47.2	47.6	0.01
Overweight and obesity	46.1	23.2	0.49*	46.0	46.5	0.01
Mental and behavioral disorders due to psychoactive substance use	46.0	29.0	0.36*	45.9	46.4	0.01
Diabetes mellitus	27.7	14.6	0.32*	27.7	28.1	0.01
Chronic ischemic heart disease	17.7	7.5	0.31*	17.6	18.3	0.02
Post-traumatic stress disorder (PTSD)	15.3	11.6	0.11*	15.2	15.1	<.001
Heart failure	14.5	4.8	0.33*	14.5	14.5	<.001
Persons with potential health hazards related to socioeconomic and psychosocial circumstances	14.3	11.3	0.09	14.3	14.2	<.001
Pre-existing suicidal ideations	13.9	14.8	0.03	13.8	13.8	<.001
Epilepsy and recurrent seizures	6.4	3.2	0.15*	6.3	6.7	0.02
Schizophrenia, schizotypal, delusional, and other non-mood psychotic disorders	5.8	4.0	0.08	5.7	5.6	0.01
Cerebral infarction	5.7	2.8	0.14*	5.7	6.0	0.01
Personal history of self-harm	5.7	6.0	0.01	5.6	5.4	0.01
Intentional self-harm	2.4	1.3	0.08	2.3	2.5	0.01
Pre-existing suicide attempt	2.1	1.5	0.04	2.0	1.9	0.01
